# The relationship between erythema nodosum and prognosis in systemic sarcoidosis: a retrospective cohort study^☆^

**DOI:** 10.1016/j.abd.2021.09.011

**Published:** 2022-07-07

**Authors:** Elif Afacan Yıldırım, Perihan Aladağ Öztürk, Esra Adışen, Nurdan Köktürk

**Affiliations:** a Department of Dermatology, Faculty of Medicine, Gazi University, Ankara, Turkey; b Department of Pulmonary Medicine, Faculty of Medicine, Gazi University, Ankara, Turkey

**Keywords:** Erythema nodosum, Prognosis, Sarcoidosis

## Abstract

**Background:**

Erythema Nodosum (EN) is the most common skin manifestation in sarcoidosis and has often been associated with a good prognosis.

**Objectives:**

To compare the clinical characteristics and treatment-related features in patients with sarcoidosis according to whether or not EN was seen as a presenting symptom at the time of diagnosis.

**Methods:**

A 20-year single-center retrospective study was performed. The following two groups were identified: one group with EN as one of the presenting symptoms at the time of diagnosis of sarcoidosis (EN group) and a second group without EN as a presenting symptom at diagnosis (non-EN group). The clinical characteristics and treatment modalities were collected from the medical records.

**Results:**

A total of 122 patients (31 in the EN group, 91 in the non-EN group) were included. Radiological stages of pulmonary disease were significantly lower in the EN group. Articular involvement was more common in the EN group (p = 0.001), whereas other systemic organ involvements (p = 0.025), especially neurological involvement (p = 0.036), were significantly more common in the non-EN group. In the EN group, a higher percentage of patients were managed without systemic therapy (71.0% vs. 54.9%) and spontaneous remission was more frequent (25.0% vs. 14.1%), however, this wasn’t statistically significant.

**Study limitations:**

Retrospective design.

**Conclusions:**

The lower radiological stage of pulmonary sarcoidosis and lower frequency of systemic organ involvement in patients with EN augment the prognostic value of EN highlighted in the literature. However, this study couldn’t confirm that the patients with EN would need less systemic therapy in the course of their disease.

## Introduction

Sarcoidosis is a multisystemic disease of unknown etiology that is characterized by the formation of granulomas in different organ systems. It was first described in 1877 and since then great progress has been made in elucidating the clinical, immunological, and pathological aspects of the disease.^1^ The etymology of sarcoid goes back to the Greek word sarkodes meaning ‘fleshy or flesh-like’ and in 1899, Norwegian dermatologist Caesar Boeck used the term sarcoid to define multiple skin nodules resembling sarcoma.^2^, ^3^

The prevalence of sarcoidosis is 4.7‒64 in 100.000 people and the incidence is 1‒35.5 in 100.000 people per year with the highest frequency observed in Northern European and African-American individuals.^4^, ^5^ In Turkey, the estimated annual incidence of sarcoidosis is reported as 4 in 100.000 people.^6^ The disease is slightly more common in women and the difference is more pronounced in African-Americans with a female to male ratio of almost 2:1.^7^, ^8^ The onset of sarcoidosis peaks during the third decade of life, and 70% of patients are between the ages of 25‒45.^1^, ^5^

Cutaneous involvement in sarcoidosis varies between 9%‒37% in different studies. 1, 9, 10, 11 In nearly one-third of the cases skin lesions were reported among the initial findings.^12^, ^13^

Skin lesions in sarcoidosis are categorized as follows: specific lesions with histologically confirmed noncaseating granulomas and non-specific lesions which occur as a reactive process in the absence of granuloma formation.^14^ The specific skin lesions include papules and plaques, lupus pernio, angiolupoid sarcoidosis, scar sarcoidosis, subcutaneous nodules (Darier-Roussy sarcoid), and rare manifestations as hypopigmented areas, alopecia, nail findings, and sarcoid granulomas on mucous membranes.^14^ The most common non-specific skin manifestation is Erythema Nodosum (EN), reactive inflammatory panniculitis that is present in up to 25% of sarcoidosis patients.^14^, ^15^ On the other hand, although EN itself is associated with a myriad of etiologies, approximately 10%‒22% of the cases are related to sarcoidosis.^16^ Early studies indicate that EN is associated with a good prognosis in sarcoidosis.^9^, ^14^, ^17^, ^18^

In this study, the authors retrospectively analyzed patients with systemic sarcoidosis. The purpose of this study is to compare the demographic and clinical characteristics, involvement of different organ systems, and treatment-related features in patients with sarcoidosis according to whether or not EN is seen as a presenting symptom at the time of diagnosis. Thereby, the authors aim to find out whether the present data can augment the existing data on disease characteristics and prognosis related to EN in patients with sarcoidosis.

## Materials and method

In this retrospective cohort study, the authors reviewed the patients diagnosed with sarcoidosis between 1994 to 2013 in a tertiary referral medical center in Ankara, Turkey. This study was approved by the Institutional Review Board of the University Hospital. The study was conducted in accordance with the ethical standards described in an appropriate version of the 1975 Declaration of Helsinki, as revised in 2018.

The diagnosis of sarcoidosis was established by the pulmonologists according to the American Thoracic Society (ATS) statement^1^ and based on the clinical characteristics, radiological features, and histopathological evidence of noncaseating epithelioid granulomas in any organ system. The possible other causes of granulomatosis were excluded before the final diagnosis. Organ involvement was defined in accordance with the World Association of Sarcoidosis and Other Granulomatous Diseases (WASOG) organ assessment instrument.^19^

The present study’s sample of 122 patients was divided into the following two groups: one group with EN as one of the presenting symptoms at the time of diagnosis of sarcoidosis (EN group) and the second group without EN as a presenting symptom at the time of diagnosis (non-EN group).

In the studied center, all the medical information is centrally registered in an electronic data repository by the clinicians. The medical records of all patients including demographic data, clinical characteristics, diagnostic methods, and treatment modalities were collected. A systemic evaluation including pulmonary, skin, ophthalmologic, articular, neurologic, cardiac, renal, hepatic, stomach, and extra-thoracic lymph node involvement was applied to each patient. Constitutional symptoms and organ involvements at presentation were recorded.

Radiographical staging for pulmonary sarcoidosis from Stage 0 to Stage 4 was done according to a posteroanterior chest radiograph.^1^, ^5^ Tuberculin skin test and laboratory examinations including Erythrocyte Sedimentation rate (ESR) and serum Angiotensin-Converting Enzyme (ACE) levels were among the other parameters evaluated.

The statistical analyses were performed using IBM SPSS Statistics Version 23.0 (Statistical Package for Social Sciences, SPSS Inc., Armonk, NY, USA). Demographical data and disease characteristics were analyzed using descriptive statistics. Continuous variables were presented as mean ± Standard Deviation (SD), and categorical variables as frequency counts and percentages. Categorical variables were analyzed using Pearson’s Chi-Squared test. To compare the means of normally distributed continuous variables, and independent-sample *t*-test was performed and for the non-parametric data, the Mann-Whitney *U* test was used. The significance threshold was set at p-values < 0.05.

## Results

Between 1994 to 2013, a total of 122 patients were diagnosed with sarcoidosis. Of the 122 patients, 31 had EN as a presenting symptom at the time of diagnosis and constituted the EN group. The remaining 91 patients without EN as a presenting symptom were included in the non-EN group.

Female predominance was observed in both groups. A total of 83.9% (n = 26) of the patients in the EN group and 68.1% (n = 62) of the patients in the non-EN group were female. In the EN group, patient age at the time of diagnosis ranged from 18 to 61 years, with a mean ± SD of 44.4 ± 12.06. In the non-EN group, patient age ranged from 22 to 82, with a mean ± SD of 47.3 ± 12.82. However, the difference in the age of onset was not statistically significant (*t* = 1.12, p = 0.262).

Pulmonary sarcoidosis was present in all patients. A posteroanterior chest radiograph was abnormal in 98.4% (n = 120) of the patients at diagnosis. The remaining 2 patients with a normal posteroanterior chest radiograph (Stage 0), had abnormal findings in thoracic computed tomography examination. Radiological stages in each group were summarized in Table 1.

**Table 1:** Radiological stage at diagnosis in each group.

Radiological stage	EN group, n (%)	Non-EN group, n (%)
Stage-0 Normal chest radiograph	1 (3.2)	1 (1.1)
Stage-1 Lymphadenopathy alone	19 (61.3)	36 (39.6)
Stage-2 Pulmonary infiltration with lymphadenopathy	10 (32.2)	42 (46.1)
Stage-3 Pulmonary infiltration without lymphadenopathy	1 (3.2)	11 (12.1)
Stage-4 Pulmonary fibrosis	0 (0.0)	1 (1.1)

χ^2^(1) = 6,153 and p = 0.013.

Overall, the pulmonary stage was lower in EN-group and the results of the chi-square test for trend indicated a statistically significant difference, χ^2^(1) = 6,153 and p = 0.013. In correlation with this, cough, the main symptom of pulmonary sarcoidosis, was more common in the non- EN group (61.5% vs. 38.7%, p = 0.027).

In the non-EN group, 5 patients had specific cutaneous lesions of sarcoidosis with histopathological evidence of noncaseating granulomas, one of which had lupus pernio and four had red-brown papules and plaques. In the EN group, none of the patients had sarcoidosis-specific skin lesions.

Characteristics of systemic involvements other than pulmonary involvement were summarized in Table 2. There was a statistically significant difference in terms of articular involvement between the EN group and non-EN group (48.4% vs. 17.6%; p = 0.001). On the other hand, patients in the non-EN group had significantly more neurological involvement (13.2% vs. 0.0%; p = 0.036). Although the ocular, hepatic, splenic, cardiac, stomach, and extra-thoracic lymph node involvements were more common in the non-EN group, the difference was not statistically significant when each was evaluated separately. However, when these 2 groups were compared according to the presence of any systemic organ involvement other than joints, it was observed that there was significantly more organ involvement in the non-EN group (25.3% vs. 6.5%, p = 0.025).

**Table 2:** Characteristics of involvement of different organ systems.

Systemic involvements	EN group, n (%)	Non-EN group, n (%)	p
Articular	15 (48.4)	16 (17.6)	0.001
Ocular	2 (6.5)	13 (14.3)	NS
Neurological	0 (0.0)	12 (13.2)	0.036
Extra-thoracic lymph	1 (3.2)	5 (5.5)	NS
Nodes			
Hepatic	0 (0.0)	4 (4.4)	NS
Splenic	0 (0.0)	3 (3.3)	NS
Cardiac	0 (0.0)	2 (2.2)	NS
Stomach	0 (0.0)	2 (2.2)	NS
Renal	0 (0.0)	0 (0.0)	NA
Any organ involvement	2 (6.5)	23 (25.3)	0.025
Other than articular			

NS, Not Significant; NA, Not Applicable.

Constitutional symptoms like fatigue, weight loss, fever, and night sweats were also questioned. Of note, fatigue was present in 12.9% (n = 4) of the patients in the EN group and 22.0% (n = 20) in the non-EN group, fever in 12.9% (n = 4) in the EN group and 7.7% (n = 7) in the non-EN group, weight loss in none of the patients in EN group and 11.0% (n = 10) in the non-EN group, night sweats in 3.2% (n = 1) in EN group and 9.9% (n = 9) in the non-EN group. In terms of constitutional symptoms, none of the differences above were statistically significant.

When the smoking history of the patients was evaluated, the following results were obtained: in the EN group 83.9% (n = 26) of the patients were lifelong non-smokers, 12.9% (n = 4) were ex-smokers and 3.2% (n = 1) was an active smoker; in the non-EN group, 70.3% (n = 64) were lifelong non-smokers, 22.0% (n = 20) were ex-smokers and 7.7% (n = 7) were active smokers.

The level of Angiotensin-Converting Enzyme (ACE), which is a known marker for sarcoidosis and whose levels correlated with the amount of whole-body granuloma, was evaluated in 30 patients in the EN group and 70 patients in the non-EN group. Of these, the mean ± SD was 65.4 ± 61.13 and 68.1 ± 47.55, respectively, and the difference was not statistically significant. As a marker of systemic inflammation, the Erythrocyte Sedimentation Rate (ESR) was also measured in 29 patients in the EN group and 81 patients in the non-EN group with mean ± SD of 27.2 ± 16.21 and 28.3 ± 23.89, respectively, with no significant difference between them. A tuberculin skin test was applied to 21 patients in the EN group and 64 patients in the non-EN group, and 42.9% and 51.6% of them were anergic, respectively.

A total of 29.0% (n = 9) of the patients were treated with systemic treatment in the EN group, whereas 71.0% (n = 22) did not receive systemic treatment. In the non-EN group, 45.1% (n = 41) underwent systemic therapy and 54.9% (n = 50) did not. The percentage of patients who did not receive any systemic treatment was higher in the EN group; however, the difference between the two groups was not statistically significant. Oral corticosteroids were the most frequently preferred treatment option. The details of the treatment regimens in each group were summarized in Table 3. Of note, the patient who received a combination of oral corticosteroids, azathioprine, pentoxifylline and infliximab had an aggressive disease with the involvement of multiple organs including the liver, spleen, stomach, parotid gland, and also lupus pernio, a sarcoidosis-specific skin lesion.

**Table 3:** Systemic treatment regimens preferred in each group.

Systemic therapy	EN group, n (%)	Non-EN group, n (%)	p
None	22 (71.0)	50 (54.9)	0.117
Oral corticosteroids alone	5 (16.1)	34 (37.4)	0.029
Oral corticosteroids + azathioprine	1 (3.2)	4 (4.4)	NS
Oral corticosteroids + pentoxifylline	3 (9.7)	2 (2.2)	NS
Oral corticosteroids + azathioprine + pentoxifylline + infliximab	0 (0.0)	1 (1.1)	NS

NS, Not Significant.

A total of 95 patients were evaluated regarding spontaneous remission. Of these, 71 have belonged to the non-EN group and 24 were to the EN group. Spontaneous remission was observed in 25.0% (n = 6) of the patients in the EN group and in 14.1% (n = 10) in the non-EN group. Even though spontaneous remission was more frequent in the EN group, the difference was not statistically significant.

## Discussion

Cutaneous lesions are common in sarcoidosis and the skin is the second most frequently involved organ after the lungs.^21^ Skin findings in sarcoidosis often manifest as initial presenting symptoms and in fact, one study reported that cutaneous lesions developed before or at the time of diagnosis of systemic sarcoidosis in 80% of the patients.^22^ In the present study sample, 29.5% of the patients had cutaneous involvement at the time of diagnosis with EN in 25.4% and specific lesions in 4.1%. This result is consistent with the past studies reporting skin manifestations in 25%‒30% of patients with systemic sarcoidosis.^23^, ^24^ Similarly, in a cohort of Turkish patients, Yanardag et al.^12^ reported skin involvement as an initial finding in 30% of the patients and observed EN in 20.8%. The female preponderance in the present study sample (83.9% in the EN group and 68.1% in non-EN) is in line with the previous literature concerning different ethnic groups.^25^ Past research suggests a low prevalence of tobacco smoking in patients with sarcoidosis.^26^, ^27^ According to a case-control study of 706 patients, lifelong nonsmokers were more likely to develop sarcoidosis and the odds ratio was 0.65 in ever-smokers.^28^ In the present study, the prevalence of ever-smoking cigarettes was also low in both groups (16.1% in the EN group and 29.7% in the non-EN group).

In sarcoidosis, serum ACE levels are known to be increased in 60% of patients^29^ and correlate with the granuloma burden as it is produced by epithelioid cells in granulomas.^20^, ^30^ However, serum ACE level was not associated with a prognostic predictive value in the literature^31^ and the authors also could not observe a significant difference between the two groups in the present study.

EN in sarcoidosis is reported to have a prognostic significance in the course of the disease.^9^ EN is characterized by erythematous-violaceous tender nodules on the extensor aspects of the lower legs and has often been associated with a good prognosis in previous studies.^9^, ^14^, ^17^, ^18^^,^^21^ The significantly lower radiological stage of pulmonary sarcoidosis in patients with EN in the present study population supports the prognostic value of EN highlighted in previous studies as the higher stage of chest radiography is associated with a worse prognosis.^32^ Nearly half of the patients (48.4%) with EN had an articular involvement in the present study and this was significantly more common than the non-EN group (17.6%). This finding was in correlation with the well-known coexistence of EN and arthralgia/arthritis in Lofgren syndrome, an acute presentation of sarcoidosis.^14^, ^33^ It is characterized by the association of EN with bilateral hilar lymphadenopathy, arthralgia, and fever in patients with sarcoidosis.^14^ In relation to this, fever was the only constitutional symptom that was more common in the EN group in the present study’s sample, even though the difference was not statistically significant. Patients with Lofgren syndrome were reported to remit spontaneously in more than 80% of the cases.^1^ It should be noted that the existence of all three cardinal features (EN, acute polyarthritis and bilateral hilar lymphadenopathy) is not required for a diagnosis of Lofgren syndrome.^14^

Regarding organ involvement, neurological involvement was significantly less common in patients with EN (0.0% vs. 13.2%, p = 0.036) in the present study’s cohort, and to our knowledge, this has not been previously mentioned in the literature. Strikingly, when all the systemic organ involvements other than articular findings were compared between the two groups, there was significantly more organ involvement in the non-EN group (25.3% vs. 6.5%, p = 0.025) and this result may be interpreted as EN indicating a good prognosis in sarcoidosis. In the present study, the percentage of patients who received systemic therapy was lower in the EN group (29.0% vs. 45.1%); however, the difference was not statistically significant. Therefore, although the present authors of this study agree that systemic organ involvement is less common in patients who had EN as an initial presenting symptom, the authors couldn’t confirm that these patients would need less systemic therapy in the course of their disease. Future studies with larger populations are needed to further investigate this relationship.

## Limitations

The present study has some limitations. First, it is a retrospective study based on the centrally registered medical records of the patients. The present study has a single-center design with a limited number of patients, thus the number of patients with certain systemic organ involvements was too small to analyze statistically. Furthermore, since the follow-up time varies between the patients, the authors could not interpret the final outcomes in each group with systemic sarcoidosis.

## Conclusion

Sarcoidosis is a multi-organ disease with protean clinical manifestations. Since the skin findings in sarcoidosis, especially EN, often appear as initial symptoms, dermatologists involve in the management of the disease from the very beginning. The lower radiological stage of pulmonary sarcoidosis and lower frequency of organ involvement, in particular neurological involvement, in patients with EN in the present study augment the prognostic value of EN highlighted in the literature. However, the present authors of this study could not confirm that these patients would need less systemic therapy in the course of their disease.

## Financial support

None declared.

## Authors’ contributions

Elif Afacan Yıldırım: Concept, design, definition of intellectual content; literature search; data acquisition and data analysis, manuscript preparation; manuscript editing.

Perihan Aladağ Öztürk: Concept, data acquisition and data analysis, manuscript preparation.

Esra Adışen: Concept, design, definition of intellectual content, final approval of the final version of the manuscript.

Nurdan Köktürk: Effective participation in the research guidance of intellectual content; manuscript review, final approval of the final version of the manuscript.

## Conflicts of interest

None declared.
